# Parent satisfaction with sustained home visiting care for mothers and children: an integrative review

**DOI:** 10.1186/s12913-022-07666-3

**Published:** 2022-03-03

**Authors:** Kie Kanda, Stacy Blythe, Rebekah Grace, Lynn Kemp

**Affiliations:** 1 School of Nursing and Midwifery, Translational Research and Social Innovation Group, Western Sydney University, Ingham Institute for Applied Medical Research, 1 Campbell Street, Liverpool, NSW 2170 Australia; 2 Transforming Early Education and Child Health, Translational Health Research Institute, Western Sydney University, Campbelltown, NSW 2560 Australia

**Keywords:** Parent satisfaction, Satisfaction, Home visiting, Maternal-child health services, Integrative review

## Abstract

**Aim:**

To synthesise and analyse the existing literature regarding parent satisfaction with sustained home visiting care for mothers and children.

**Background:**

Sustained home visiting is a service delivery mechanism of both prevention and intervention, in which people receive structured support services within their home environment over an extended period of months or years. For the purposes of this paper, sustained home visiting refers to in-home nursing support to address health inequities for mothers and young children. Sustained home visiting programs have been found to support improved health, wellbeing, and developmental outcomes for children and families. However, there is limited knowledge with regards to the level of parent satisfaction with care provided at home, and the factors and elements of care parents perceive to be critical to their satisfaction. It is important for healthcare practitioners to understand what practices and process parents consider to be a priority in securing their ongoing engagement.

**Design:**

Integrative review.

**Data sources:**

PubMed/Medline, CINAHL, Embase, and PsycINFO.

**Methods:**

A multi-step approach was used to search and retrieve peer-reviewed studies from the databases. Study selection, data extraction, data synthesis and critical appraisal were undertaken by two independent researchers.

**Results:**

A total of 13 studies met the inclusion criteria, including nine quantitative and four qualitative studies. The review found that parents provided with home visiting interventions had higher levels of satisfaction with care than those who received routine or facility-based care. Service dose was a factor associated with parent satisfaction, however, the direction of impact on parent satisfaction was mixed. Other elements of care parents perceived as important to service satisfaction included the nurse-client relationship, being treated with respect, empowerment, and emotional support.

**Conclusion:**

While it is critically important that home visiting practitioners provide evidence-based care and interventions, it is equally important that services are delivered in the context of positive and empowering relationships. Further research is recommended to understand the care process and mechanisms that enhance parent satisfaction and positive experiences, providing optimal quality of care.

**Supplementary Information:**

The online version contains supplementary material available at 10.1186/s12913-022-07666-3.

## Background

Previous research in early human development demonstrates the importance of the first one thousand days of life after conception to positive and life-long child outcomes [1, 2]. This body of research calls for evidence-based early intervention services within this sensitive window of time, targeting parents, caregivers and children who have been identified as at risk of poor outcomes [3]. Sustained home visiting (SHV) programs have been a critical part of this service response, supported by a strong evidence base. This paper provides a review of the existing research as this relates to parent satisfaction with the delivery of SHV programs.

Sustained home visiting is a service delivery mechanism and a form of both prevention and intervention that has been employed to improve a range of maternal and child health, well-being, and education outcomes [4, 5]. The major aim of the SHV programs is to promote health equity through a focus on mothers and families experiencing adversity by delivering multiple services in the family’s home environment in an intensive and sustained structure extended over months or years, predominantly, predominantly provided by nurses and midwives [6]. Such programs have been implemented widely in various countries such as the US, the UK, Australia, and New Zealand [7–9]. Since the 1980s, there has been increasing research evidence to support the effectiveness of SHV programs. In particular, these interventions have been found to support positive outcomes for children and families [10–14], including: increased maternal parenting confidence; improved parenting knowledge; positive home environments to support healthy child development [9, 15, 16]; improved access to welfare services [17]; and decreased rates of child physical abuse, neglect and the criminal behaviour of parents [18–21]. While there is a strong body of research demonstrating program effectiveness, there is limited knowledge on the mechanisms that support positive change for families, the practices and processes that contribute to positive outcomes and the characteristics of the clients who benefit most.

In this review, we focused on parent satisfaction with the care provided in sustained nurse home visiting, as satisfaction is crucial to the provision of effective interventions and positive experiences for mothers and children. There are several frameworks and models which underpin client satisfaction and quality of care. Underpinning this review is the WHO framework for the quality of maternal and newborn healthcare. The WHO conceptualisation of quality of care is described as having two dimensions: provision and experience of care [22]. For effective provision of care, there needs to be clear articulation of which components and elements of care are essential to positive outcomes based on existing research evidence [23]. Experience of care consists of effective communication, respect and preservation of dignity, and emotional support [22]. Both dimensions are essential to providing quality care which is client-centred and evidence-based.

Client satisfaction is one of the most widely accepted outcome indicators reflecting the quality of health care systems as it offers information on the provider’s success at meeting clients’ values and expectations [24–29]. Donabedian (2005) argues that client satisfaction is the ultimate outcome of health care [24]. It is also considered a nursing-sensitive client outcome significantly impacted by nursing interventions [30]. Client satisfaction provides insightful measures of client perceptions of the care processes and their sense of empowerment resulting from increased knowledge, understanding and improved capacity for managing health and well-being [31]. Higher satisfaction with health care services changes client behavioural intentions, such as compliance with recommended treatment which results in better health outcomes [32]. Furthermore, previous research indicated that client satisfaction is related to engagement, retention, and completion [33–36]. However, family engagement remains a challenge for most of the SHV programs [8, 37]. Thus, it is essential for practitioners to understand what practices and process parents consider to be a priority in securing their ongoing engagement [38]. Assessing the perceptions, views and preferences of mothers and families about their experiences of maternal, newborn and early childhood health care is considered a research priority globally [39].

In this integrative review, we aimed to summarise and synthesise the existing literature to provide an understanding of parent satisfaction with SHV care. The guiding review questions were: “What is the level of parent satisfaction with SHV care delivered at home by nurses or midwives?” and “What are the factors and elements of care that parents find important for satisfaction with SHV care by nurses or midwives?”. Answering these questions may provide new insights to improve parent satisfaction and quality of care in SHV interventions which promote optimal outcomes for mothers and families.

## Methods

### Design

An integrative review is a specific review method that summarises past empirical or theoretical literature to provide a more comprehensive understanding of a particular phenomenon or healthcare problem [40]. It allows for the inclusion of diverse research designs such as quantitative, qualitative, and mixed methods designs within a single review [41–43]. As the purpose of this study was to build our understanding of nursing, particularly regarding home visiting practice, a wide-reaching review in the form of an integrative review was judged to be suitable.

The five stage methodology framework by Whittemore and Knafl [43] guided this review. It includes: (1) problem identification; (2) literature search; (3) data evaluation; (4) data analysis; and (5) presentation. This framework was developed to specifically address intricacies commonly encountered during the integrative review process such as the need to combine research from multiple study methods. We also conducted this review according to the Preferred Reporting Items for Systematic Reviews and Meta-Analysis (PRISMA) guidelines [44], as there is no specific reporting guidance for integrative reviews [45]. The review protocol was registered in the Prospective Register of Systematic Reviews (PROSPERO, Registration number CRD42020221861).

### Search methods

A systematic search of the literature was performed to identify studies focused on parent satisfaction with sustained nurse home visiting care services for mothers and children. The databases searched were PubMed/Medline, CINAHL, Embase, and PsycINFO.

The search strategy was designed to be as extensive as possible to identify all eligible studies according to the inclusion and exclusion criteria detailed below. A multi-step search approach was used to retrieve peer-reviewed studies. To be eligible for inclusion, studies need to have been published in English. We did not limit our search by publication date.

The search strategy, including all identified keywords and index terms as shown in Table 1, was adapted for each included information source in consultation with the research librarian. Different terminologies and spellings of keywords were considered to help in the identification of relevant studies. Combinations of keywords and terms using Boolean operators, truncation, phrase searching, and Medical Subject Headings (MeSHs) were used in the search strategies.

**Table 1:** Keywords used in literature search

Concept	MeSH^a^ terms or keywords
Participants	Maternal, mother, woman/women, pregnancy [MeSH], Child [MeSH], Infant [MeSH], baby/babies, newborn, early childhood
Providers	Nurs^*^ or midwife or midwives
Intervention	Sustained home visiting, Home visit/home-visit, nurse home visit, home-based, home care services [MeSH], Home health nursing [MeSH], Home Nursing [MeSH], maternal health services [MeSH],
Outcomes	Satisfaction, maternal satisfaction, parent satisfaction, client satisfaction, patient satisfaction [MeSH], experience, perception

^a^*MeSH* Medical Subject Headings, ^*^truncation symbol

Articles were selected based on the following inclusion and exclusion criteria.

i) Participants: Pregnant women, mothers, and caregivers and their children aged under five years old
ii) Intervention: Maternal and early childhood care and interventions during the antenatal, postnatal, or early childhood period, targeting pregnant women, mothers, or caregivers of young children. These care and intervention services included home visiting care and services by a nurse or midwife. Studies were excluded if participants were provided with the care and interventions at a health centre, clinic, hospital, or any other formal health care facility.
iii) Outcomes: Parent satisfaction with care delivered at home. It included satisfaction in general or satisfaction with specific aspects of care such as communication and nurse-client relationship.
iv) Types of studies: Quantitative, qualitative, and mixed methods studies.

Following the search, all studies yielded in the search were imported into Endnote X9 software and duplicates subsequently removed. The selection of studies was conducted over three stages. In the first stage, titles and abstracts were screened to include all potentially relevant studies by the first author. Then, the matrix of that literature was shared with one of the other authors (SB). In the second stage, abstracts were screened according to the eligibility criteria by two researchers (KK, SB). After this initial selection, the full texts of the remaining studies were reviewed independently by two researchers (KK, SB) for final inclusion. The remaining authors (RG, LK) were available to resolve any disagreements regarding study inclusion, however none arose. Final studies selected for full-text screening were recorded. Reasons for exclusion were documented in the selection process.

### Search outcomes

**Fig. 1:**
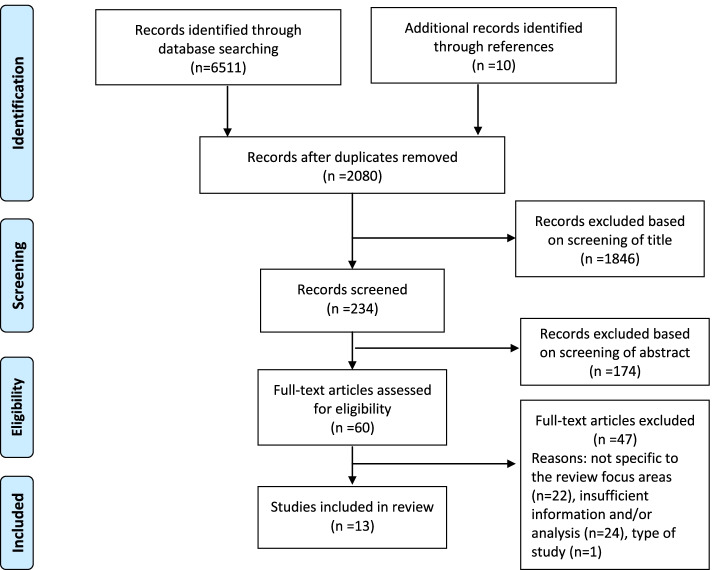
Search process and results

The search results yielded 6511 titles. After removal of duplication and irrelevant titles, 234 were identified for the abstract screening. Then, 60 were identified for the full-text screening. After the final screening, a total of 13 studies were selected for inclusion in the review as illustrated in Fig. 1. Forty-seven studies were excluded for the following reasons: (1) not specific to the review area: participants (*n* = 11); providers (*n* = 8); interventions (*n* = 3); (2) insufficient information and/or analysis undertaken such as no results on satisfaction included (*n* = 24); and (3) type of study (*n* = 1).

### Quality appraisal

The Mixed Methods Appraisal Tool (MMAT) version 2018 [46] was used to assess the quality of all the included studies. The MMAT evaluates the appropriateness of the study aim, study design methodology, recruitment of participants, data collection, data analysis, results presentation, discussion by authors and conclusion. However, no studies were excluded on the basis of the overall quality scores as it is discouraged by Hong and colleagues [46], and also in order to have a comprehensive summary of the existing evidence on parent satisfaction with home visiting care and interventions.

### Data extraction and synthesis

Data were extracted by the first author using a structured form and checked for accuracy by another author. The data extracted included title, author, year of publication, country of study, setting, study population, study design, aims and objectives, analytic method, intervention, and parent satisfaction. Data were collated, summarised, and reported using text and table.

The analytic method employed a narrative synthesis approach [47]. This is an approach to systematically review and synthesise findings from multiple studies that relies primarily on the use of words and text to summarise and explain the finding of the synthesis [47]. A narrative summary of the results from quantitative and qualitative studies (Table 3) was generated using a matrix, where the included studies were presented to identify parent satisfaction and the elements of care that parents found important for satisfaction. Furthermore, the findings from each study were presented in two focus areas. The first focus area looked at the level of satisfaction with care provided in sustained home visiting interventions. The second focus area explored the key factors and elements of care related to parent satisfaction across the included studies. The first author independently examined all the studies to identify the reoccurring key terms from the research findings. Simultaneously, another author examined the studies. The two authors then discussed and reached consensus on the key terms.

## Results

### Description of the studies

In total, 13 studies met the inclusion criteria as presented in Table 2. Of the 13, nine were quantitative studies and four were qualitative studies. There were no mixed-methods studies that met the inclusion criteria. The studies took place in five countries; Australia (*n* = 6), the United States of America (USA; *n* = 2), Germany (*n* = 2), Canada (*n* = 2), and Ireland (*n* = 1).

**Table 2:** Characteristics of studies included in the review

Author, Year	Country	Study design	Participants	Frequency of home visits, intervention duration, and providers
***Quantitative studies***				
**Kemp et al., 2019** [8]	Australia	Randomised controlled trial	*N* = 352 Women < 37 weeks gestation, having sufficient English proficiency, having two or more of ten sociodemographic risk factors by screening	Intended frequency: Antenatal—fortnightly, birth to 6 weeks—weekly, 1.5 to 3 months—fortnightly, 6 months to 2 years—bi-monthly Duration: antenatal to 2 years Providers: nurses
**Goldfeld et al., 2018 **[48]	Australia	Randomised controlled trial	*N* = 722 (Intervention = 363, Control = 359) Women < 37 weeks gestation, having sufficient English proficiency, having two or more of ten sociodemographic risk factors	Intended frequency: Antenatal—fortnightly, birth to 6 weeks—weekly, 1.5 to 3 months—fortnightly, 6 months to 2 years—bi-monthly Duration: antenatal to 2 years Providers: nurses
**Fraser et al., 2000 **[49]	Australia	Randomised controlled trial	*N* = 181(Intervention = 90, Comparison = 91) Mothers at risks (one of the risk of sole parent, ambivalence, domestic violence, childhood abuse, OR three or more of eight sociodemographic risks)	Intended frequency: Weekly visits until the infant was 6 weeks old, fortnightly visit until the infant was 3 months old, then monthly visit until the age of 12 months Duration: after birth to 1 year Providers: nurses
**Armstrong et al., 1999 **[50]	Australia	Randomised controlled trial	*N* = 181(Intervention = 90, Comparison = 91) Mothers at risks (one of the risk of sole parent, ambivalence, domestic violence, childhood abuse, OR three or more of eight sociodemographic risks)	Intended frequency: Weekly visits from the primary visiting nurse until the child age 6 weeks, fortnightly visit until the child age 3 months, monthly visit until the child age 12 months Duration: after birth to 1 year Providers: nurses
**Armstrong et al., 2000 **[51]	Australia	Randomised controlled trial	*N* = 160(Intervention = 80, Comparison = 80) Mothers at risks (one of the risk of sole parent, ambivalence, domestic violence, childhood abuse, OR three or more of eight sociodemographic risks)	Intended frequency: Weekly visits from the primary visiting nurse until the child age 6 weeks, fortnightly visit until the child age 3 months, monthly visit until the child age 12 months Duration: after birth to 1 year Providers: nurses
**Christie & Bunting, 2011 **[52]	Ireland	Randomised controlled trial	*N* = 295 (Intervention = 136, Control = 159) First-time low risk mothers	Intended frequency: Six home visits from 10 – 14 days to 8 weeks postpartum (weekly home visit). Families were followed-up at 7 months postpartum Duration: after birth to 8 weeks Providers: nurses
**Bashour et al., 2008 **[53]	Syria	Randomised controlled trial	*N* = 876 3 groups: 4 postnatal visits (*n* = 285), one visit (*n* = 294), or no visit (*n* = 297)	Intended frequency: One or four postnatal visits on day 3 (for one visit) and day 1, 3, 7, and 30 after childbirth (for four visits) Providers: midwives
**Brand & Jungmann, 2014 **[34]	Germany	Cross-sectional study	*N* = 434 Low-income, first-time mothers	Intended frequency: Weekly in the first four weeks of program participation, bi-weekly after that until child ages 2 years Duration: Antenatal period (12–28 weeks pregnant) to child ages 2 years Providers: midwives
**Brand & Jungmann, 2012 **[54]	Germany	Quasi‐experimental study	*N* = 430 Low-income, first-time mothers	Intended frequency: Weekly in the first four weeks of program participation, bi-weekly after that until child ages 2 years Duration: Antenatal period (12–28 weeks pregnant) to child ages 2 years Providers: midwives
***Qualitative studies***				
**Zapart et al., 2016 **[55]	Australia	Qualitative descriptive study	*N* = 36	Reported frequency: On average, participants received 20 home visits of the program’s 25 scheduled visits Duration: Antenatal to 104 weeks after birth Providers: nurses
**DeMay, 2003 **[56]	USA	Qualitative study (content analysis)	*N* = 62, Intervention group = 22, current practice group = 40	Reported frequency: Intervention group received almost twice the average number of visits per year (8 and 4.8 respectively) and twice the average amount of time per visit (120 and 60 min, respectively) as current practice group clients Duration: no specific description Providers: nurses
**Landy et al., 2012 **[57]	Canada	Qualitative case study	*N* = 18 Low income, young (< = 21 years), and first-time mothers	Reported frequency: biweekly except during the first month of the intervention and the first postpartum month, when nurses visit weekly Duration: Before 29 weeks gestation until the child age 1 year Providers: nurses
**Byrd, 1998 **[58]	USA	Qualitative descriptive study	*N* = 11 (One nurse and 10 clients)	Reported frequency: every one to two weeks Duration: no specific description, but data collection took 8 months Providers: nurse

Of the nine quantitative studies, seven were randomised controlled trials with sample sizes ranging from 160 to 876 participants. Other quantitative studies included one quasi-experimental design and one cross-sectional design. The majority of participants in the included studies were parents with sociodemographic risks such as low income, unemployment, first-time mothers, and limited time in formal education. The primary purpose of these quantitative studies was to assess the effectiveness of home visiting programs and inventions.

All four qualitative studies employed a narrative study design. The primary purpose of these studies was to describe parents’ perceptions and experiences of sustained home visiting care. Two of the qualitative studies used in-depth and semi-structured interviews, one used both interviews and observations, and one drew data from essays written by parents for data collection.

### Intervention characteristics and measures of parent satisfaction

All interventions were home visiting programs for women or mothers and children. In six studies [8, 34, 48, 54, 55, 57], interventions commenced in the antenatal period and continued up to the postpartum period. The interventions were provided by nurses (*n* = 10) and midwives (*n* = 3).

All quantitative studies employed questionnaires or simple survey instruments to capture information on parent satisfaction. Four studies [48–51] used the tools modified from the Parent Satisfaction Questionnaire (PSQ), and others used the Service-surgery Satisfaction Questionnaire (*n* = 1), and the Session Rating Scale (SRS) (*n* = 1). The remaining studies (*n* = 3) used author-constructed questionnaires for data collection. Queries ranged from binary ‘yes/no’ questions to multiple point Likert scales for scoring the levels of parent satisfaction. Satisfaction with specific elements of care, such as communication, convenience, interpersonal manner, and time spent, as well as overall satisfaction, were asked in the questionnaires in most of the studies.

In three qualitative studies [55–57], women were interviewed about their perceptions and experiences of home visiting care and interventions. One study [56] undertook a content analysis of essays (*n* = 62) written by mothers about their experiences in nurse home visiting program.

### Level of parent satisfaction with sustained home visiting care

In all but one of the quantitative studies [53], parents reported higher levels of satisfaction with home visiting interventions compared to routine or standard community care or facility-based services, as shown in Table 3.

**Table 3:** Narrative synthesis matrix on satisfaction with care provided and measures used

Author, Year	Findings on satisfaction	Measures used
Kemp et al., 2019 [8]	•Families in the intervention rated the Session Rating Scale (SRS) highly with the mean score 39.4 (SD 1.31) out of possible 40	Session Rating Scale (SRS)
Goldfeld et al., 2018 [48]	•The scores of families’ Parent Satisfaction Questionnaire (PSQ) were rated more highly by the families in the intervention program than usual care: higher PSQ scores of the intervention group (mean = 44.4, SD = 4.1) out of possible 50 with compared to the usual care group (mean = 37.9, SD = 7.2) (*p* < 0.001)	Parent Satisfaction Questionnaire (PSQ) (modified)
Fraser et al., 2000 [49]	•Greater satisfaction for the home visiting program group compared with comparison group participants accessing standard clinic-based services •Statistically significant group differences (home visiting group compared with standard clinic-based services group) were found for each sub-scale of satisfaction: communication (*p* < 0.05); convenience (*p* < 0.05); interpersonal manner (*p* < 0.05); general satisfaction (*p* < 0.05); time spent (*p* < 0.05); and overall satisfaction (*p* < 0.05) with greater satisfaction reported for the home visiting program	A short form of the PSQ III. Measures of technical quality and financial aspects were excluded, using only the statements of communication, convenience, interpersonal manner, general satisfaction, and time spent. Overall satisfaction was calculated by addition of these five subscales
Armstrong et al., 1999 [50]	•Statistically significant group differences were found for every scale used to measure satisfaction with the home-based program at 6 weeks	Modified PSQ-18. Ten items from the PSQ-18 were used. The measures of technical quality and financial aspects were excluded. Ten statements for general satisfaction, interpersonal manner, communication, time spent, and accessibility/convenience
Armstrong et al., 2000 [51]	•Statistically significant group differences were found for every scale used to measure satisfaction with the service at 4 months, greater satisfaction being shown by those in the home-based program: communication (*p* < 0.05), manner (*p* < 0.05), satisfaction (*p* < 0.05), time spent (*p* < 0.05), overall satisfaction (*p* < 0.05)	Modified PSQ-18. Ten items from the PSQ-18 were used. The measures of technical quality and financial aspects were excluded. Ten statements for general satisfaction, interpersonal manner, communication, time spent, and accessibility/convenience
Christie & Bunting, 2011 [52]	•The itervention group had higher service satisfaction (mean = 154.6, SD = 23.8) (out of possible 170) with compared to the control group (mean = 139.9, SD = 29.7) (*p* < 0.000) at 8 weeks; and (mean = 150.4, SD = 27.6) and (mean = 134.2, SD = 37.5) (*p* = 0.003) respectively at 7 months	Service-surgery satisfaction questionnaire
Bashour et al., 2008 [53]	•The answer to the question “you are happy about your experience during the postnatal period”. The percentage responded ‘Yes’ was 80.1% among mothers received 4 postnatal visits, 81.0% among mothers received 1 postnatal visit, and 84.2% among mothers received no visit	A questionnaire constructed by researchers
Brand & Jungmann, 2014 [34]	•With regards to process variables, unsuccessful visits attempt, maternal engagement during home visits, quality of the helping relationship, satisfaction with service and time spent on parenting issues during pregnancy were associated with both early and late attrition. Satisfaction with service Odds Ratio (OR) = 0.56 (95% Confidential Interval (CI) 0.45–0.70) (*p* < 0.001)	An author-constructed questionnaire with 4-item scale:1) dissatisfied, 2) rather dissatisfied, 3) rather satisfied, 4) satisfied
Brand & Jungmann, 2012 [54]	•Ratings of satisfaction with service and quality of the relationship were higher in the comparison model (both *p* < 0.05, but unequal variances)	Satisfaction with service was measured on an author-constructed, 4-item scale: 1) dissatisfied, 2) rather dissatisfied, 3) rather satisfied, 4) satisfied
Zapart et al., 2016 [55]	•27 participants were happy with the structure of the program, that is, what happened during the visits, and did not think any changes were needed. Program length was talked about by 17 women. Five were happy with the program concluding the child-age 2- years, ten thought it should run for longer. Only two women thought the program should run for less than 2 years •28 participants talked about their relationship with the nurse. 24 mothers described it as being good to excellent or saying they got on very well. Women stated that the nurse was ‘very friendly’, ‘very nice’, ‘non-judgemental’, and ‘straightforward’	N.A
DeMay, 2003 [56]	•Participants were satisfied with learning from the nurse on how to understand their infant better. “I think the nurses do an excellent job encouraging good eating habits, trying to avoid stress and making you aware of this wonderful little person living inside you, and how its future depends on you” “It’s just amazing to me to see what goes on in an infant’s life” •Participants wrote about how information helped relieve feelings of being unprepared, afraid, and anxious during their pregnancy •Descriptions demonstrated how much they valued the intimate relationship with their public health nurse	N.A
Landy et al., 2012 [57]	•Mothers’ experiences in the program were very positive and highlight the critical importance of the nurse-client relationship •The positive relationships described by the mothers had multiple dimensions which are captured under the following six subthemes: (1) the nurse’s personality; (2) The nurse is “like a friend” who supports you; (3) the nurse is respectful and trusting; (4) the nurse is empowering and an advocate; (5) the nurse is an honest expert; and (6) the nurse is easy to access when you need her help	N.A
Byrd, 1998 [58]	•A participant stated, “I like the support and help the nurse gives me. It is wonderful. Someone to validate you”. Likewise, another mother stated, “She says ‘You’re doing right’ because sometimes I question it. Am I doing the right thing? Yeah. She’s been good” •A participant described that the nurse was familiar with the characteristics and needs that participant mothers shared	N.A

Goldfeld and colleagues [48] reported that family satisfaction with services was rated more highly by the home visiting intervention group than the usual care group. Similarly, Kemp and colleagues [8] showed that families who received the home visiting intervention rated the Session Rating Scale (SRS) more highly than the usual care group. There was no evidence of differences in SRS scores at any time between those who completed the program (SRS mean = 39.5, SD = 1.2) and those intervention families who did not complete the program (SRS mean = 38.9, SD = 1.9).

Fraser and colleagues [49] reported that the sustained home visiting intervention group reported significantly greater satisfaction with the care received compared with the comparison group who received standard clinic-based services. Furthermore, there were significant differences for sub-scales of communication, convenience, interpersonal manner, general satisfaction, time spent, and overall satisfaction with greater satisfaction for the intervention group. Similarly, the study conducted by Armstrong and colleagues [51] showed statistically significant differences for every scale used to measure satisfaction with the service at four months, with greater satisfaction for the home visiting intervention group than standard community health services.

In contrast, only one study (*n* = 876) reported a lower percentage (80.1%) of home visiting intervention group mothers who were satisfied with their experience compared with the comparison group (84.2%) who received services in health facilities [53]. The authors of this study hypothesised that the lower level of satisfaction among the intervention group was because the midwives who delivered the service were recruited from a hospital setting, rather than a community setting. Thus, they may not have had the competencies necessary for effective home visiting service delivery, such as advanced listening and empathy skills, or experience in engaging with individualised problem-solving techniques.

Zapart and colleagues [55] reported that of 36 participating women, 27 were satisfied with the structure of the program, that is, what happened during the visits, and did not think any changes were needed. Program length was discussed by 17 participants. Five were satisfied with the program concluding when the child was two years old, and ten thought it should run for a longer period of time. Only two women stated the program should run for less than the standard two years. Similarly, Landy and colleagues [57] found that mothers’ experiences with home visiting were positive. Most of the mothers shared the view that they were becoming better parents by participating in the program [57]. In a study conducted by DeMay [56], the mothers were satisfied with learning from a nurse how to understand their infant better.

### Factors and elements of care that parents find important for satisfaction in sustained home visiting care

None of the studies included in this review were specifically designed to identify the factors and elements of care that were important for parent satisfaction with sustained home visiting care. However, data analysis and synthesis provide insight into factors associated with parent satisfaction and critical elements of care that could contribute to parent satisfaction: service dose; nurse-client relationship; care with respect and empowerment; and emotional support.

In several studies [8, 52, 53], service dose was tested for its association with parent satisfaction with the care provided at home. Christie and Bunting [52] conducted a study to determine the effect of the service dose (frequency of home visits). The study results showed that mothers who received six postpartum visits reported higher levels of satisfaction with the care provided compared to mothers who received only one visit at eight weeks and another at seven months. Similarly, Bashour and colleagues [53] found that a larger number of home visits was associated with positive service experience [53]. In addition, one qualitative study [57] suggested a positive relationship between satisfaction and program retention. However, one study showed no relationship between program completion and satisfaction [8].

All four qualitative studies included in this review [55–58] reported how much women valued the intimate relationship with home visiting nurses. Landy and colleagues [57] found that mothers’ accounts highlight the critical importance of the nurse-client relationship. The positive relationships described by the participant mothers had multiple dimensions, including: the nurse’s personality; the nurse is ‘like a friend’ who supports them; the nurse is respectful and trusting; the nurse is empowering and an advocate; the nurse is an honest expert; and the nurse is easy to access when they need help. Similarly, a study conducted by DeMay [56] also demonstrated how much the participant mothers valued the intimate relationship with their home visiting nurse. This study highlighted the importance of consistency in the relationship and found an association between best outcomes for clients with nurse consistency throughout the program. In the study conducted by Zapart and colleagues [55], 28 of the 36 participants talked about their relationship with the nurse, and 24 described the relationship as being good to excellent. The participating women described the nurses as ‘very friendly’, ‘very nice’, ‘non-judgmental’, and ‘straightforward’. Byrd [58] described client-nurse relationships as two-way, easy, conformable, relaxed, informal and friendly across home visits.

The mothers valued being treated with respect and empowered to make their own choices in home visiting care. Mothers found information and care helpful when it was provided in a respectful and non-judgmental manner [56]. One woman stated that ‘I think the nurses do an excellent job encouraging good eating habits, trying to avoid stress and making you aware of this wonderful little person living inside you, and how its future depends on you”. Participants also talked about how information given by a nurse helped relieve feelings of being unprepared, afraid, and anxious during their pregnancy [56].

The participants in a study by Byrd [58] described feeling strengthened by the nurse who provided emotional support and expressed admiration for mothers in their caregiving efforts. A foster mother who participated in the study stated that “I like the support and help the nurse gives me. It is wonderful. Someone to validate you”. Likewise, another mother said “she (nurse) says ‘You are doing right’ because sometimes I question it – am I doing the right thing? Yeah, she has been good”.

## Discussion

The purpose of this review was to synthesise the research relating to parent satisfaction with home visiting care and interventions for mothers and children. Knowledge of critical factors and elements of care that are important for parent satisfaction in sustained home visiting care is extremely important to the provision of more effective interventions and positive experiences for mothers and children.

Overall, this review found that in all but one study, women who were provided with home visiting services showed greater satisfaction with the care they received, compared to women who received services in clinic settings. The findings also indicate high levels of parent satisfaction with specific aspects of service delivery as this relates to communication, convenience, and interpersonal manner. This may be because SHV services provide opportunities for practitioners to observe the environments in which families live, which can help them identify a family’s unique needs and provide a greater level of individual attention than is possible in usual facility-based care (Goldfeld et al., 2018) [48]. Furthermore, such services are provided by a designated nurse for the extended period. A study that compared the competencies of generalist nurses and home visiting nurses stated that the language of the generalist nurse is one of structure, power, and control: of assessing, monitoring, and controlling a client who is absent or passive in the discourse. In contrast, SHV nursing competency requires a less controlling language of participation and cooperation with the client, and focus on strengths [59]. These differences in competencies may explain the higher level of parent satisfaction with the communication and interpersonal manner of home visiting nurses.

This review identified several elements of care which contribute to parent satisfaction. Nurse-client relationship was considered a critical factor in parent satisfaction. In previous research, the effectiveness of the home visiting intervention has been attributed to the practitioners’ development of therapeutic relationships with clients [17]. Home visiting values an effective relationship between practitioners and mothers to help mothers reach their goals. Mothers choose to enrol for different reasons and intentions, such as getting information about child development and specific assistance [60, 61]. A better relationship is established in the process that practitioners respond to these reasons and needs in various ways [62]. Furthermore, effective relationships between practitioners and parents require practitioners to support mothers to become empowered, active participants who make healthy choices for themselves and their families [63]. In addition, previous studies have indicated that mothers with better relationship with home visitors were more likely to have higher levels of program involvement and complete the program [33, 64].

Engaging in the care process with respect and empowerment, and an providing emotional support were elements of relationship that were seen as critical for parent satisfaction. As described in the studies conducted by Zapart and colleagues [55] as well as DeMay [56], nurses who are perceived as non-judgmental are better able to create safe environments that facilitate the building of trusting relationships [63]. These care processes are compatible with the WHO framework for the quality of care of maternal and newborn health care [22]. Effective communication, respect and preservation of dignity, and emotional support are essential to positively impactful client experiences.

### Implications for nursing practice

This review suggested the critical importance of nurse-client relationship, care with respect and empowerment, and emotional support in home visiting care. Previous evaluation research supports that parenting programs are most effective when trusting relationships have been established between professionals and parents [65]. It is important for home visiting care practitioners to understand that, as important as it is to provide evidence-based essential care and services for parents and children, is it equally important that these services are provided in a respectful and empowering manner. The provision of appropriate and meaningful emotional support is a critical element of satisfaction with home visiting care. Thus, there is a need for capacity building amongst the workforce to enhance knowledge of how to provide appropriate emotional support to vulnerable families.

### Implications for future research

There are several implications for future research in the SHV services. First, as this review found that the relationship between service dose and satisfaction was mixed, there is a need for more research to explore the relationship between dose and parent outcomes. Second, this review revealed the fact that the measurement of parent satisfaction is often not built into study design, though it is an important indicator of effectiveness for SHV programs. Thus, further research is required to understand the practices, processes, and mechanisms associated with parent satisfaction and other key outcomes of home visiting interventions for mothers and children. We propose the need for research which is designed to explore service characteristics that drive higher satisfaction and better outcomes. For example, there is a need for research that examines if the customised care practices and processes employed in the care provided to mothers and families, based on their individual and unique circumstances, needs, and preferences, and the relationship between this customisation and parent satisfaction. This research would focus on variations in care, and the decision-making processes of practitioners that sits behind program adaptations by listening to and observing mothers and families. Such research evidence will inform strategies to improve further the quality of care based on the clients’ voices, view and experiences. We believe that more in-depth qualitative or mixed-methods research will deepen knowledge in this area.

### Strengths and limitations

This review had several limitations. First, all studies meeting the inclusion criteria were incorporated in the review regardless of the quality of the research design or methods. Although this allowed for a comprehensive summary of the evidence on parent satisfaction with home visiting care and interventions, some findings might have limited validity. Second, this review included only peer-reviewed studies published in the English language. Studies published in other languages may have more diverse results on parent satisfaction in different cultural contexts. Third, although the search strategy was designed to find all potentially relevant articles, some studies might have been missed. Despite these limitations, this integrative review sheds light on an important gap in the research about parent satisfaction with home visiting care. This review identified only 13 studies, which means that parent satisfaction is underreported in the literature. Thus, there is still much to understand about the factors related to parent satisfaction and elements of care which parents consider important to service satisfaction.

## Conclusion

This is the first review synthesising and analysing parent satisfaction, which is considered one of the critical outcomes of home visiting programs. In all but one study, parents provided with home vising care and interventions delivered by community-based practitioners reported a higher level of satisfaction with care than those who received routine or facility-based services. The review found that there is little knowledge of the elements that underpin parent satisfaction with care provided at home, for example, the practices and process that determine and promote parent satisfaction, other than an explicit finding on the importance of the nurse-client relationship, care with respect and empowerment, and emotional support. We still have much to understand about the process and mechanisms involved in the care provided at home to support improved client experiences and satisfaction, ultimately leading to positive child and family outcomes.

## Supplementary Information

**Additional file 1.**

## Data Availability

Data sharing is not applicable to this article since all data are retrievable from the original sources. Articles included in the review are also summarised in Table 2.

## Abbreviations

SHV: Sustained home visiting
PRISMA: Preferred Reporting Items for Systematic Reviews and Meta-Analysis
PROSPERO: Prospective Register of Systematic Reviews
MeSHs: Medical Subject Headings
MMAT: The Mixed Methods Appraisal Tool
PSQ: Parent Satisfaction Questionnaire
SRS: Session Rating Scale
