# Supplementary material for: Parent satisfaction with sustained home visiting care for mothers and children: an integrative review
Source: BMC Health Serv Res. 2022 Mar 3;22:295. doi: 10.1186/s12913-022-07666-3 (PMC8895511; doi:10.1186/s12913-022-07666-3)
Supplement: Supplementary file 1 — Additional file 1. [file 12913_2022_7666_MOESM1_ESM.docx]

**Table (supplemental). Quality assessment of included studies using Mixed Methods Appraisal Tools (MMAT)**

|  | **Qualitative studies** | | | | | **Randomised controlled trials** | | | | | **Quantitative descriptive studies** | | | | |
| --- | --- | --- | --- | --- | --- | --- | --- | --- | --- | --- | --- | --- | --- | --- | --- |
| **Author/s, Year** | **1.1.** | **1.2.** | **1.3.** | **1.4.** | **1.5.** | **2.1** | **2.2.** | **2.3.** | **2.4.** | **2.5.** | **4.1.** | **4.2.** | **4.3.** | **4.4.** | **4.5.** |
| Kemp et al., 2019 | - | - | - | - | - | - | - | - | - | - | Yes | Yes | Yes | Yes | Yes |
| Goldfeld et al., 2018 | - | - | - | - | - | Yes | Yes | Yes | Yes | Yes | - | - | - | - | - |
| Fraser et al., 2000 | - | - | - | - | - | Yes | Yes | Yes | Yes | Yes | - | - | - | - | - |
| Armstrong et al., 1999 | - | - | - | - | - | Yes | Yes | Yes | Yes | Yes | - | - | - | - | - |
| Armstrong et al., 2000 | - | - | - | - | - | Yes | Yes | Yes | Yes | Yes | - | - | - | - | - |
| Christie & Bunting, 2011 | - | - | - | - | - | Yes | Yes | Yes | Yes | Yes | - | - | - | - | - |
| Bashour et al., 2008 | - | - | - | - | - | Yes | Yes | Yes | Yes | c.t. | - | - | - | - | - |
| Brand & Jungmann, 2014 | - | - | - | - | - | - | - | - | - | - | Yes | Yes | Yes | Yes | Yes |
| Brand & Jungmann, 2012 | - | - | - | - | - | - | - | - | - | - | Yes | Yes | Yes | Yes | Yes |
| Zapart et al., 2016 | Yes | Yes | Yes | Yes | Yes | - | - | - | - | - | - | - | - | - | - |
| DeMay, 2003 | Yes | Yes | Yes | Yes | Yes | - | - | - | - | - | - | - | - | - | - |
| Landy et al., 2012 | Yes | Yes | Yes | Yes | Yes | - | - | - | - | - | - | - | - | - | - |
| Byrd, 1998 | Yes | c.t. | Yes | Yes | Yes | - | - | - | - | - | - | - | - | - | - |

Note: the items of the MMAT which were not applicable for the assessment in this review (3. Non-randomised studies and 5. Mixed methods studies) were removed from the table. Abbreviation: c.t. - Can’t tell.
